# Effect of Abdominal Weight Training on Diaphragm Thickening Fraction and Respiratory Muscle Strength in Healthy People: A Quasi-Experimental Study

**DOI:** 10.7759/cureus.95429

**Published:** 2025-10-26

**Authors:** Kakeru Hasegawa, Ayano Sai, Kazuki Okura, Masahiko Satake

**Affiliations:** 1 Division of Rehabilitation, Akita University Hospital, Akita, JPN; 2 Department of Rehabilitation Medicine, Nagamachi Hospital, Sendai, JPN; 3 Department of Physical Therapy, Akita University School of Health Sciences, Akita, JPN

**Keywords:** abdominal weight training, diaphragm, quasi-experimental, respiratory muscle training, ultrasound imaging

## Abstract

Introduction

Abdominal weight training (AWT) is a simple technique that applies external resistance to the diaphragm during breathing by placing weights on the abdomen. Although it is an accessible method for respiratory muscle training, its physiological effects remain unclear. This quasi-experimental study in healthy young adults evaluated the immediate and four-week effects of AWT on diaphragm thickening fraction (DTF) and maximal inspiratory pressure (MIP).

Methods

A quasi-experimental study was conducted with 26 healthy participants allocated to either an AWT group or a control group. The AWT group performed diaphragmatic breathing with abdominal weights for four weeks, while the control group performed unloaded diaphragmatic breathing. DTF was assessed under different weight conditions to examine immediate effects, and both DTF and MIP were measured on days 0, 14, and 28 to evaluate longitudinal effects using mixed-effects models.

Results

No significant immediate effect of abdominal loading on DTF was observed (β = 0.15; 95% confidence interval: -3.81 to 4.10; *p* = 0.94), with DTF decreasing under the 5-kg load condition. Over the four-week intervention, no significant group × time interaction was found for DTF (β = -0.13; 95%CI: -0.51-0.26; *p* = 0.53) or MIP (β = -0.00; 95%CI: -0.17-0.17; *p* = 0.99). However, both groups showed modest increases in DTF and MIP over time.

Conclusion

AWT did not significantly improve DTF or respiratory muscle strength compared to unloaded diaphragmatic breathing in healthy young adults. These findings suggest that AWT, in its current form, may have limited utility, and optimization of training parameters is necessary before broader application. Key limitations include a small sample, non-random allocation, and a short four-week period; longer, optimized trials are warranted.

## Introduction

Ultrasound enables noninvasive assessment of the diaphragm. Diaphragm thickness (DT) and diaphragm thickening fraction (DTF) are practical indices of diaphragmatic function. Increases in DT correlate with vital capacity and maximal inspiratory pressure (MIP), whereas reductions in DT are associated with diminished inspiratory strength [1,2]. Therefore, DT and DTF can be used to monitor load and responsiveness during simple respiratory interventions such as abdominal weight training (AWT).

The respiratory muscle complex, including the diaphragm, intercostal muscles, and abdominal muscles, is essential for breathing [3,4]. Diaphragmatic weakness reduces ventilatory efficiency and may limit everyday activities. Respiratory muscle training (RMT) is used across diverse conditions and generally improves respiratory muscle strength, including MIP and maximal expiratory pressure (MEP) [5-7]. The standard approach to inspiratory muscle training (IMT) uses threshold-loading devices [8]; however, reliance on such devices can limit accessibility in some settings.

AWT is a simple method in which several kilograms of weight are placed on the abdomen during diaphragmatic breathing to provide external resistance. Its simplicity and independence from specialized equipment make AWT a viable option for clinical practice and home-based self-training. However, evidence regarding optimal loading parameters and the immediate and long-term effects of AWT remains limited, and standardized techniques have not yet been established. Mechanistically, AWT elevates intra-abdominal pressure via abdominal muscle activation driven by diaphragmatic pressure differences, thereby increasing the mechanical work performed by the diaphragm during inspiration. When appropriately dosed, this loading may foster task-specific adaptations in diaphragmatic function and inspiratory muscle performance [6].

This study aimed to investigate the effects of a four-week AWT regimen on DTF and MIP in healthy young adults, providing useful information for optimizing training loads and developing standardized AWT protocols. We hypothesized that a four-week AWT program, using a weight load optimized for each individual’s highest baseline DTF, would considerably improve DTF and MIP.

## Materials and methods

Study design

This was a non-randomized quasi-experimental study conducted at the Akita University Hospital, Akita City, Akita Prefecture, Japan. The assessor was blinded to group allocation, whereas participant blinding was not feasible due to the nature of the intervention. The study was approved by the Ethics Committee of the Akita University Graduate School of Medicine (approval number: 2927). All participants received written and verbal information about the study and provided written informed consent prior to data collection.

Participants

Healthy young adults from the local community were recruited via poster advertisements. Eligibility criteria included age 20-40 years, non-smoking status, no acute respiratory infection within the previous four weeks, ability to perform diaphragmatic breathing in the supine position, and sufficient comprehension to follow instructions and provide consent. Participants were excluded if they had a history of chronic respiratory disease (e.g., chronic obstructive pulmonary disease (COPD), asthma) or cardiac disease, neuromuscular, thoraco-abdominal, or spinal disorders that could affect breathing, inability to lie supine due to kyphosis or vertebral compression, current abdominal or lower-back pain, or prior major thoraco-abdominal surgery, were pregnant, or refused/withdrew informed consent.

Sample size

No a priori power calculation was performed. Consistent with the Transparent Reporting of Evaluations with Nonrandomized Designs (TREND) statement, we prespecified a pragmatic target sample size based on anticipated recruitment during a predefined window (December 2023-March 2024) and operational considerations (personnel availability and ultrasound assessment capacity).

Study protocol

Following baseline measurements, participants were alternately assigned to either the AWT or the control group.

Breathing Training Structure

Participants lay supine and performed diaphragmatic breathing from residual volume (RV) to total lung capacity (TLC). Each daily session comprised two sets of 30 breaths for four weeks. Breathing cadence was metronome-paced at approximately six to eight breaths/minute; on each breath, participants executed a five-second end-inspiratory breath-hold at TLC, followed by relaxed expiration. Seated rest of one to two minutes was provided between sets.

AWT Protocol

At baseline, DT was measured under six randomly ordered loads (0-5 kg); the single load that yielded the maximal DTF was selected and kept constant throughout the program (no progression). Weights were placed symmetrically adjacent to the midline at the 10th costal margin, in a fabric pouch secured to prevent shifting. The control group performed the same breathing exercises without added weight. When feasible, a researcher supervised sessions in person and provided real-time feedback. DTF, respiratory function, and inspiratory muscle strength were assessed on days 14 and 28.

Outcome measures

Diaphragm Thickening Fraction

DT was measured using a portable ultrasound system (SONIMAGE MX1, Konica Minolta, Inc., Chiyoda, Tokyo, Japan) in B-mode with a linear 10-MHz transducer and fixed presets (gain/depth/focus). All ultrasound assessments were performed by a single trained assessor blinded to group allocation. In the supine position, the probe was placed on the right hemidiaphragm at the zone of apposition between the anterior and mid-axillary lines at the 8th-9th intercostal space [9]. Minimal probe pressure was applied to avoid tissue compression. Measurements were obtained at end-expiration (RV) and end-inspiration (TLC), each repeated three times with ≥30 second rest; means were analyzed. For baseline assessment, DT was measured under six randomly ordered weight conditions (0-5 kg). DTF (%) was calculated as: $DTF (\%) = \frac{DT_{TLC} - DT_{RV}}{DT_{RV}} \times 100$. Intra-rater reliability was ICC = 0.92.

Inspiratory Muscle Strength

MIP was measured with a handheld respiratory pressure meter (Autospiro AS-507; Minato Medical Science Co., Ltd., Osaka, Japan) in accordance with European Respiratory Society (ERS) recommendations [10]. Participants were seated upright, wearing a nose clip and mouthpiece, performed a maximal inspiratory effort from RV, and maintained the plateau for approximately three seconds. Three trials were obtained with ≥30 second rest; the highest value (cmH₂O) was recorded. Measurements were conducted by the blinded assessor.

Respiratory Function

Spirometry was performed using an electronic spirometer (Autospiro AS-507) following ERS standards. Participants were seated and nose-clipped; at least three acceptable maneuvers were obtained, and the best values for forced vital capacity (FVC), forced expiratory volume in one second (FEV₁), and the FEV₁/FVC ratio were analyzed.

Demographic Data

Age and sex were recorded; height and weight were measured by study staff using standard procedures, and BMI was calculated.

Statistical analysis

Data are presented as mean ± standard deviation (SD) or median (interquartile range (IQR)), as appropriate. Baseline between-group comparisons used unpaired t-tests, Mann-Whitney U tests, or chi-square tests. The acute effect of weight loading on DTF during loaded breathing was evaluated using a mixed-effects model with DTF as the dependent variable, weight load (0-5 kg) as a fixed effect, and subject as a random effect. The longitudinal effects of AWT on DTF and inspiratory muscle strength were assessed using mixed-effects models including group allocation and baseline values as fixed effects, subject as a random effect, and a group × time interaction. All models employed restricted maximum likelihood estimation. No missing data occurred. At the primary time point (Day 28), we report adjusted between-group differences with 95% confidence intervals (CIs) and standardized effect sizes (Cohen’s d and Hedges’ g with small-sample correction). Effect sizes were computed by dividing the adjusted difference by the pooled baseline (time 0) standard deviation of each outcome (MIP or DTF). Standard errors and 95% CIs for effect sizes were obtained by scaling the model-based SE by the same denominator and using the model’s degrees of freedom (Kenward-Roger approximation for mixed-model inferences). All tests were two-sided with p < 0.05. Analyses were performed in R version 4.4.2 (R Foundation for Statistical Computing, Vienna, Austria).

## Results

Participant characteristics

Participant demographics and baseline characteristics are summarized in Table 1. Twenty-six individuals were enrolled, with 13 assigned to the AWT group and 13 to the control group. In the AWT group, the optimal load weights were 1 kg (n = 2), 2 kg (n = 1), 3 kg (n = 6), and 4 kg (n = 5). No significant between-group differences were observed in demographic variables, DTF, respiratory muscle strength, or respiratory function (Table 1).

**Table 1:** Baseline characteristics of the participants by group Note: Data presented as n (%) for sex; mean ± SD for age, weight, BMI, FVC, FEV1, and FEV1/FVC; and median (25th percentile, 75th percentile) for DT, DTF, and MIP. AWT, abdominal weight training; BMI, body mass index; DT, diaphragm thickness; DTF, diaphragm thickening fraction; FEV1, forced expiratory volume in one second; FVC, forced vital capacity; MIP, maximal inspiratory pressure

Variables	Overall (n = 26)	AWT group (n = 13)	Control group (n = 13)	p value
Sex (male), n (%)	10 (38%)	6 (47%)	4 (31%)	0.69
Age (years), mean ± SD	21.4 ± 0.9	21.5 ± 1.0	21.4 ± 0.9	0.83
Weight (kg), mean ± SD	57.0 ± 10.3	58.3 ± 12.2	55.7 ± 8.4	0.53
BMI (kg/m^2^), mean ± SD	21.0 ± 2.9	20.9 ± 2.9	21.3 ± 1.5	0.88
DT (mm), median (IQR)	1.25 (1.09, 1.40)	1.25 (1.09, 1.50)	1.25 (1.15, 1.33)	0.52
DTF (%), median (IQR)	141.4 (103.5, 176.7)	157.4 (140.6, 191.2)	115.9 (94.7, 138.7)	0.06
MIP (cmH_2_O), median (IQR)	71.3 (59.9, 91.4)	71.7 (62.8, 90.7)	67.2 (55.1, 92.7)	0.47
FVC (% predicted), mean ± SD	89.4 ± 13.6	90.4 ± 11.3	88.2 ± 16.6	0.66
FEV_1_ (% predicted), mean ± SD	86.3 ± 7.2	87.3 ± 8.7	85.4 ± 5.6	0.51
FEV_1_/FVC (%), mean ± SD	86.7 ± 9.9	87.6 ± 10.6	87.8 ± 7.3	0.61

Acute effects of weight loading

**Figure 1:**
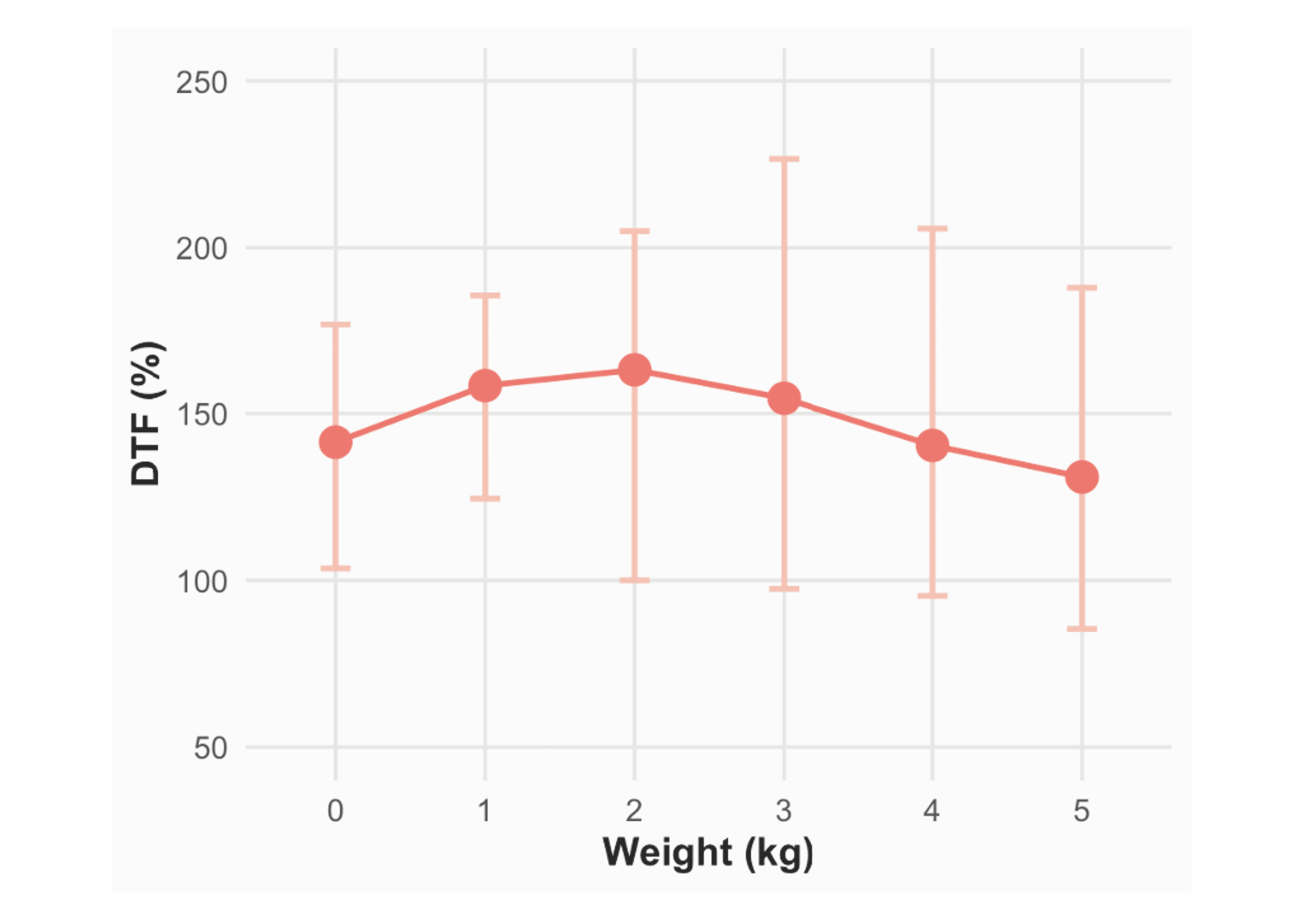
DTF across abdominal weight loads (0–5 kg) Mean DTF values are plotted for each weight condition (0, 1, 2, 3, 4, and 5 kg). Dots represent the estimated marginal means of DTF from a mixed-effects analysis, and vertical whiskers indicate the 95% CIs derived using restricted maximum likelihood estimation. The dashed line at the 0 kg condition denotes the baseline DTF. Although the 5 kg load shows a lower mean DTF than the baseline value, the CIs overlap, indicating no statistically significant immediate effect of weight loading (β = 0.15; 95%CI: –3.81 to 4.10; p = 0.94). CI, confidence interval; DTF, diaphragm thickening fraction

Mixed-effects analysis showed no statistically significant acute effect of weight loading on DTF (β = 0.15; 95%CI: -3.81 to 4.10; p = 0.94) (Table 2). DTF values under each load condition are illustrated in Figure 1. Notably, DTF measured under the 5-kg load was lower than that measured without loading.

**Table 2:** Acute effect of abdominal weight loading on DTF Note: Data are presented as β (estimate), SD, 95% CI (2.5%, 97.5%), and p value from a mixed-effects analysis with DTF as the dependent variable, weight load (0–5 kg) as a fixed effect, and subject as a random intercept. DTF, diaphragm thickening fraction; SD, standard deviation; CI, confidence interval

Outcome	Factor	β	SD	2.5%	97.5%	p value
DTF	weight	0.15	2.01	-3.81	4.10	0.94

Longitudinal effects of AWT

**Figure 2:**
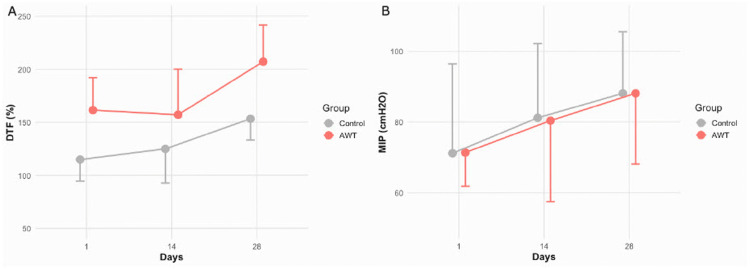
Trajectories of DTF and MIP over four weeks by group Line graphs depict mean values (±95% CIs) of each outcome at baseline (day 0), mid-intervention (day 14), and post-intervention (day 28) for the AWT group (solid circles and solid lines) and control group (open squares and dashed lines). (A) DTF: A modest upward trend in DTF was observed in both the AWT and control groups over time. However, no significant difference in the rate of change was detected between groups (group × time interaction: β = –0.13; 95% CI: –0.51 to 0.26; p = 0.53), suggesting that abdominal weight loading did not enhance diaphragmatic function beyond that achieved through diaphragmatic breathing alone. (B) MIP: Both groups exhibited slight increases in MIP throughout the intervention. However, no significant between-group difference was observed over time (β = –0.00; 95% CI: –0.17 to 0.17; p = 0.99), indicating that AWT had no additional effect on inspiratory muscle strength. CI, confidence interval; DTF, diaphragm thickening fraction; MIP, maximal inspiratory pressure; AWT, abdominal weight training

Mixed-effects analysis revealed no significant group × time interaction for any outcome (Table 3). No interaction effect between AWT and time was detected for DTF (β = −0.13; 95%CI: −0.51 to 0.26; p = 0.53) or MIP (β = 0.00; 95% CI: −0.17 to 0.17; p = 0.99). Longitudinal changes in each outcome are summarized in Table 4 and Figure 2.

**Table 3:** Mixed-effects analysis of DTF and MIP over four weeks Note: Estimates are presented as β (estimate), SD, 95% CI (2.5%, 97.5%), and p value from a mixed-effects analysis in which DTF or MIP served as the dependent variable; fixed effects included group (AWT vs. control), time (day 0, 14, 28), their interaction (AWT × time), and the respective baseline outcome value; subject was included as a random intercept. AWT, abdominal weight training; DTF, diaphragm thickening fraction; MIP, maximal inspiratory pressure; SD, standard deviation; CI, confidence interval; *, indicates p < 0.05; **, indicates p < 0.01.

Outcome	Factor	β	SD	2.5%	97.5%	p value
DTF	AWT × time	-0.13	0.20	-0.51	0.26	0.53
	AWT	0.34	0.37	-0.37	1.04	0.37
	time	0.29	0.13	0.04	0.54	0.03*
MIP	AWT × time	-0.00	0.09	-0.17	0.17	0.99
	AWT	-0.01	0.15	-0.29	0.28	0.96
	time	0.19	0.06	0.06	0.31	< 0.01**

**Table 4:** Changes in DTF and MIP across baseline, day 14, and day 28 by group Note: Values are presented as median (25th percentile, 75th percentile) at baseline, day 14, and day 28 for each group. No inferential statistics are shown here; between‐group comparisons at each time point are reported in the main text. AWT, abdominal weight training; DTF, diaphragm thickening fraction; MIP, maximal inspiratory pressure; IQR, interquartile range

Outcome	AWT group (n = 13)			Control group (n = 13)		
	baseline, median (IQR)	day 14, median (IQR)	day 28, median (IQR)	baseline, median (IQR)	day 14, median (IQR)	day 28, median (IQR)
DTF (%)	157.4 (140.6, 191.2)	133.45 (106.3, 172.7)	177.2 (145.9, 233.9)	115.9 (94.7, 138.7)	100.0 (89.1, 189.3)	193.3 (130.0, 241.7)
MIP (cmH_2_O)	71.4 (61.9, 89.0)	80.4 (57.5, 106.0)	88.1 (68.1, 106.1)	71.2 (58.8, 95.6)	74.0 (56.8, 100.2)	90.0 (75.9, 110.4)

## Discussion

This study examined both the immediate and four-week effects of AWT on DTF and MIP in healthy young adults. AWT did not result in immediate improvements in DTF; instead, DTF decreased under the 5-kg load condition. Furthermore, the four-week intervention did not yield significant improvements in DTF or MIP compared to diaphragmatic breathing alone. To our knowledge, this is the first interventional study to systematically assess DTF across multiple loading conditions and directly evaluate the efficacy of AWT. Strengths of this study include a controlled, comparative design with repeated assessments at immediate and four-week time points, the use of objective physiological outcomes (DTF and MIP), and mixed-effects analyses appropriate for repeated measures.

The lack of immediate improvement in DTF is consistent with previous studies [11]. Similar findings in adolescents have shown minimal short-term changes in respiratory muscle strength and expiratory flow rates [12], while ultrasonography studies indicate that loaded breathing does not necessarily enhance diaphragmatic thickness or contractility [13]. The observed reduction in DTF under the 5-kg load suggests that excessive external loading may impair diaphragmatic function. During normal breathing, diaphragmatic contraction increases intra-abdominal pressure and facilitates caudal displacement of the diaphragm [14]. Excessive loading may restrict this movement, while abdominal muscle contraction during exhalation may displace the diaphragm cranially [15]. Comparable restrictions have been described in obesity, where abdominal fat accumulation impedes diaphragmatic descent and reduces TLC [16]. Monteiro et al. reported that a 6-kg abdominal load increases intra-abdominal pressure but does not exceed the fatigue seen with diaphragmatic breathing alone [17], suggesting that excessive loading may limit effective diaphragmatic recruitment.

Regarding longitudinal effects, earlier studies suggest that diaphragmatic training can improve DTF and MIP [7,11]. However, no between-group differences were detected in this study. Possible explanations include suboptimal training intensity and the relatively short four-week duration. The effectiveness of RMT depends on training type, load, and duration [18,19], indicating that future studies should explore alternative loading strategies and extended intervention periods. Importantly, non-significant between-group findings should not be construed as equivalence. Given the modest sample size, the study may have been underpowered to detect small effects; the 95%CIs therefore remain informative and indicate that small group differences cannot be excluded.

Although group-level effects of AWT were limited, both DTF and MIP improved over time regardless of loading condition. This trend suggests that enhanced awareness of breathing and feedback-based practice may have contributed to improvements in respiratory function. Prior study indicates that biofeedback-guided breathing training can improve diaphragmatic function [20], supporting this interpretation. These findings suggest that AWT may primarily facilitate conscious breathing regulation rather than directly strengthening the diaphragm. Comparisons with other modalities, such as incentive spirometry or threshold-based IMT [21], could help refine indications and optimize protocols.

This study has some limitations. First, the nonrandomized, quasi-experimental design (alternate allocation) limits causal inference and may allow residual confounding despite baseline adjustment and assessor blinding. Second, the small sample size (n = 26), single-center setting, and short intervention period (4 weeks) without longer-term follow-up reduce statistical power and generalizability and preclude conclusions about the durability of effects. Third, participants were healthy young adults; effects in populations with respiratory disease (e.g., chronic obstructive pulmonary disease) remain unknown, and respiratory muscle training may be more effective in older adults and individuals with pulmonary disease [22], which constrains generalizability to clinical populations. Fourth, training intensity may have been suboptimal: AWT loads were fixed as the weight eliciting the highest baseline DTF (no progression), and potential practice effects from repeated testing cannot be excluded for volitional measures. Accordingly, the absence of statistically significant between-group differences should not be interpreted as equivalence; adequately powered randomized trials with optimized loading (selection and progression) and longer duration are warranted.

## Conclusions

In healthy young adults, AWT did not produce acute increases in DTF; under a 5-kg load, DTF decreased compared with that with unloaded breathing. Over four weeks, AWT provided no additional benefit in DTF or MIP compared to diaphragmatic breathing. These results indicate that, under the tested conditions, external abdominal loading does not enhance diaphragmatic function and may even suppress thickening at higher loads.
